# Increased pericardial fat accumulation is associated with increased intramyocardial lipid content and duration of highly active antiretroviral therapy exposure in patients infected with human immunodeficiency virus: a 3T cardiovascular magnetic resonance feasibility study

**DOI:** 10.1186/s12968-015-0193-2

**Published:** 2015-10-31

**Authors:** Mariana Diaz-Zamudio, Damini Dey, Troy LaBounty, Michael Nelson, Zhaoyang Fan, Lidia S. Szczepaniak, Bill Pei-Chin Hsieh, Ronak Rajani, Daniel Berman, Debiao Li, Rohan Dharmakumar, W. David Hardy, Antonio Hernandez Conte

**Affiliations:** Division of Nuclear Medicine, Department of Imaging & Medicine, Cedars-Sinai Medical Center, Los Angeles, CA USA; Biomedical Imaging Research Institute, Cedars-Sinai Medical Center, Los Angeles, CA USA; David-Geffen School of Medicine, University of California Los Angeles, Los Angeles, CA USA; Department of Medicine, Cardiovascular Center, University of Michigan, Ann Arbor, MI USA; Diabetes and Obesity Research Institute, Cedars-Sinai Medical Center, Los Angeles, CA USA; Heart Institute, Cedars-Sinai Medical Center, Los Angeles, CA USA; Department of Anesthesiology, Cedars-Sinai Medical Center, 8700 Beverly Boulevard, Suite 8211, Los Angeles, CA 90048 USA

**Keywords:** Human immunodeficiency virus, HIV, Cardiovascular magnetic resonance, Spectroscopy, Pericardial fat, Highly active antiretroviral therapy, HAART, Intramyocardial lipid content

## Abstract

**Background:**

The aim of the current study was to examine whether the use of highly active antiretroviral therapy (HAART) in patients with HIV is associated with changes in pericardial fat and myocardial lipid content measured by cardiovascular magnetic resonance (CMR).

**Methods:**

In this prospective case-control study, we compared 27 HIV seropositive (+) male subjects receiving HAART to 22 control male subjects without HIV matched for age, ethnicity and body mass index. All participants underwent CMR imaging for determination of pericardial fat [as volume at the level of the origin of the left main coronary artery (LM) and at the right ventricular free wall] and magnetic resonance spectroscopy (MRS) for evaluation of intramyocardial lipid content (% of fat to water in a single voxel at the interventricular septum). All measurements were made by two experienced readers blinded to the clinical history of the study participants. Two-sample *t*-test, Spearman’s correlation coefficient or Pearson’s correlation coefficient and multivariable logistic regression were used for statistical analysis.

**Results:**

Pericardial fat volume at the level of LM origin was higher in HIV (+) subjects (33.4 cm^3^ vs. 27.4 cm^3^, *p* = 0.03). On multivariable analysis adjusted for age, Framingham risk score (FRS) and waist/hip ratio, pericardial fat remained significantly associated to HIV-status (OR 1.09, *p* = 0.047). For both HIV (+) and HIV (-) subjects, pericardial fat volume showed strong correlation with intramyocardial lipid content (*r* = 0.58, p < 0.0001) and FRS (*r* = 0.53, *p* = 0.0002). Among HIV (+) subjects, pericardial fat was significantly higher in patients with lipo-accumulation (37 cm^3^ vs. 27.1 cm^3^, *p* = 0.03) and showed significant correlation with duration of both HIV infection (*r* = 0.5, *p* = 0.01) and HAART (*r* = 0.46, *p* = 0.02).

**Conclusions:**

Pericardial fat content is increased in HIV (+) subjects on chronic HAART (>5 years), who demonstrate HAART-related lipo-accumulation and prolonged HIV duration of infection. Further investigation is warranted to determine whether increased pericardial fat is associated with higher cardiovascular risk leading to premature cardiovascular events in this patient population.

## Background

Human immunodeficiency virus (HIV) infection persists as a global pandemic despite significant advances in both therapeutic and preventive interventions. For the past 5 years in the United States, the number of new persons diagnosed with HIV infection has averaged 55,000 per year [1, 2]. While the all-cause mortality directly related to HIV infection has decreased substantially since the introduction of highly active antiretroviral therapy (HAART), an increasing number of HIV-infected persons are presenting with newly diagnosed cardiovascular disease (CVD). Morbidity and mortality related to HIV infection remains high, with an increasing number of cardiovascular events in this population [3, 4].

There is also evidence that HAART itself may contribute to cardiovascular risk, secondary to side-effects leading to lipodystrophy, hyperlipidemia, hyperglycemia and potential direct cardiotoxicity [5–7]. Epicardial and thoracic fat have been associated with numerous endpoints of CVD in non-HIV-infected populations (coronary artery calcification, adverse cardiovascular events and myocardial ischemia) [8–12]. Previous studies have found increased epicardial fat in HIV-positive subjects and have raised questions about its role in this population in elevating cardiovascular risk [13, 14]. Recently, researchers have also identified increased lipid content within the cardiac muscle (cardiac steatosis) in HIV patients, and this has also been promoted as a potential marker for cardiovascular risk in both HIV negative (-) and HIV seropositive (+) individuals [15–17].

With this background, the primary aim of the current study was to determine whether individuals with HIV on chronic HAART have increased pericardial fat as assessed by cardiovascular magnetic resonance (CMR) compared to HIV-seronegative control subjects who have not been exposed to HAART as either pre-exposure or post-exposure prophylaxis. The secondary aim was to assess whether such changes are related to traditional cardiovascular risk factors and are associated with accompanying changes in myocardial lipid content by CMR spectroscopy.

## Methods

This study was reviewed and approved by the Cedars-Sinai Institutional Review Board and all subjects provided written informed consent prior to enrollment. A Certificate of Confidentiality was obtained from the National Institutes of Health.

### Subjects

Twenty-seven HIV (+) subjects on HAART were recruited from the infectious disease clinic evaluated at the Cedars-Sinai Medical Center in Los Angeles from April 2012 through to September 2013. Criteria for inclusion for patient subjects were: male gender, age of 35–55 years, HIV-seropositive status confirmed by Western blot analysis, and continuous HAART for at least 3 years. Exclusion criteria for patient subjects included: known CVD, known CVD risk factors such as diabetes, hypertension and family history of premature CVD, history of Hepatitis C infection, intravenous drug use, prolonged interruptions of HAART (greater than 3 months), history of a current AIDS-defining illness and contraindications to undergoing magnetic resonance imaging.

### Controls

Twenty-two HIV-seronegative(-) men served as control subjects; they were recruited from the same community. HIV (-) control subjects were recruited via HIV (+) subjects’ referral of friends, significant others, acquaintances and persons in familiar social circles to approximate HIV (+) subject characteristics. Inclusion criteria included: HIV-seronegative status, age-matched (+/−3 years) of HIV (+) subjects, race matched to HIV (+) subjects, sexual orientation matched to HIV (+) subjects, social/lifestyle medical history and anthropometric characteristics [i.e. height, weight, body mass index (BMI)] were also all matched to HIV (+) subjects. HIV seronegative status of control subjects was confirmed with western blot HIV analysis prior to imaging. Exclusion criteria for control subjects included: known CVD, known CVD risk factors such as diabetes, hypertension and family history of premature CVD, history of Hepatitis C infection, intravenous drug use and any previous use of HIV-antiretroviral therapy for pre- or post-exposure prophylaxis.

### Clinical assessment

All subjects and controls completed a detailed medical and social history questionnaire. For HIV (+) subjects, detailed information regarding HIV medical history, HIV exposure history, sexual history and recreational drug use was obtained from medical records. Comprehensive HAART history was assessed by detailed review of medication records. Cumulative HAART exposure to each antiretroviral agent and class of agent was calculated based on months of exposure. Exposure to all antiretroviral agents implicated as “high risk” for CVD (i.e. abacavir, amprenavir, didanosine, fosamprenavir, indinavir, lopinavir) was also calculated.

A comprehensive physical exam was performed to assess anthropometric variables. BMI was calculated from measured height and weight. Waist/hip ratio was calculated from measured waist and hip circumference. The presence of lipodystrophy (defined as the pathologic presence or absence of adipose tissue in various anatomic locations consistent with HAART-induced side-effects) was assessed by an experienced infectious disease specialist. If lipodystrophy was present, further physical evaluation was performed to categorize (mild, moderate, severe) and to differentiate anatomic presence of lipo-accumulation (abdomen, cervico-dorsal spine, anterior neck) and/or lipoatrophy (arms, buttocks, face, legs).

A fasting venous blood sample was obtained for measurement of glucose, triglycerides, total cholesterol, HDL cholesterol, LDL cholesterol, complete blood count (hemoglobin, hematocrit, white blood cell count and differential) and basic metabolic panel (electrolytes, blood urea nitrogen and creatinine). Metabolic syndrome was defined as central obesity (waist circumference > 94 cm) plus any two of the following: raised triglycerides (> 150 mg/dL), reduced HDL cholesterol (< 40 mg/dL), elevated blood pressure (> 130/85), elevated fasting blood glucose (> 100 mg/dL) [18]. Framingham cardiac risk factor scoring (FRS) was calculated according to standard criteria, accounting for age, total cholesterol, smoking history, HDL cholesterol and systolic blood pressure [19]. Exercise levels were quantified based upon the subject’s reporting of activity as 1) no exercise activity 2) Mild = once per week 3) Moderate = 2–4 times per week and 4) High = greater than 5 times per week.

### Cardiovascular magnetic resonance protocol

All CMR and spectroscopy was performed using a 3.0-Tesla whole body scanner (MAGNETOM Vario, Siemens Medical, Erlangen, Germany). Imaging for pericardial fat content used a Turbo FLASH sequence with a multi-slice 2D transverse acquisition and single-shot per slice was performed. Thirteen slices in total, 6-mm thickness with 6-mm slice gap, FOV = 400 × 400 mm2, matrix = 144 × 192, spatial resolutio*n* = 2.78 × 2.08 × 6 mm3, flip angle = 10, TR/TE = 3.2/1.27 ms, receiver bandwidth = 651 Hz/pixel. Pericardial fat quantification was performed using commercially-available ORS Visual Imaging Software (Object Research Systems, Inc., 2013, Montreal, Canada), in the following way: 1) as volume (cm3) in a 6 mm slab at the level of the left main (LM) origin [20, 21]; 2) as volume (cm3) in a 6 mm slab at the level of the right ventricular (RV) free wall; and 3) as thickness (mm) of the pericardial fat at RV free wall in the same slice [22–24]. Tracing of the fat border was manually performed by two experienced readers in cardiovascular imaging, blinded to HIV status.

We have previously published the intramyocardial lipid content of the patients and controls studied in this investigation [16]. We included the data here for reference purposes, and to test a completely separate hypothesis (i.e. pericardial fat is related to intramyocardial lipid content). To measure intramyocardial lipid content, a spectroscopic volume of interest (single voxel, 6 cc) was positioned over the intraventricular septum using end-systolic cardiac cine images in 3 planes (short and long axis), collected at end-expiration [16, 25]. During acquisition of spectroscopic data, patients breathed freely, with spectroscopic data acquisition triggered at end-systole (via ECG gating) and end-expiration (via a respiratory navigator). Spectroscopy data were processed using commercially available software (NUTS, Acorn NMR, Fremont, CA). The areas of resonances for water and methylenes of fatty acids in triglycerides were quantified by a line-fitting procedure accounting for signal decay due to spin-spin relaxation. Triglyceride content was expressed as a percentage of fat to water.

### Statistical analysis

All continuous variables included in the analyses are presented as mean ± SD. Variables with non-normal distributions are presented as median with range. Univariate analyses were performed on continuous variables using the two-sample *t*-test for normally distributed variables and the Mann-Whitney *U* test for non-normally distributed data. The Spearman’s correlation coefficient was used to assess the relationship between continuous variables and ordinal data and the Pearson’s correlation coefficient for non-continuous variables and interval data. Multivariable logistic regression was used to determine the relationship of pericardial fat volume to HIV status using FRS, waist and hip circumference as covariates. Statistical significance for all analyses was set at the 5 % level. All statistical analyses were performed using STATA (version 10, StataCorp LP, College Station, TX).

## Results

### Patients and controls

Detailed subject demographics and lipid measurements are described in Table 1. HIV (+) subjects and HIV (-) controls were similar with regard to age, sex, anthropometric measurements and blood pressure. Resting heart rate was significantly higher in HIV (+) subjects compared to control subjects. There were no significant differences in blood glucose or lipid laboratory measurements between both groups. Seven of 27 (26 %) HIV (+) subjects and 4 of 22 (20 %) controls were taking lipid-lowering medications. Table 2 presents detailed HIV-related characteristics of the HIV (+) subjects, as well as details of their HAART exposure. All HIV (+) patients demonstrated durable immunologic restoration and persistent viral suppression for greater than 3 years and no AIDS-defining diagnoses at the time of the study.

**Table 1:** Subject characteristics

	HIV (+)	HIV (-)	p
	(*n* = 27)	(*n* = 22)	
Caucasian, %	100	100	---
Age, years	48.1 ± 5.1	48.0 ± 4.7	0.93
Weight, kg	79.7 ± 11.2	78.0 ± 13.0	0.64
BMI, kg/m^2^	25 ± 4	24 ± 4	0.34
Hip circumference, cm	96.2 ± 7.0	96.8 ± 8.7	0.80
Waist circumference, cm	90.8 ± 11.6	86.2 ± 13.1	0.22
Waist/hip ratio	0.94 ± 0.09	0.89 ± 0.10	0.07
^a^Framingham Risk Score, %	3 (4)	3 (2)	0.88
Perform regular exercise, %	85	95	0.36
Median level of exercise	2	2	---
Systolic blood pressure, mmHg	115 ± 15	122 ± 15	0.08
Diastolic blood pressure, mmHg	70 ± 9	67 ± 7	0.22
Resting heart rate, beats/min	76 ± 11	61 ± 10	<0.001
Total cholesterol, mg/dL	174 ± 33	180 ± 37	0.52
HDL Cholesterol, mg/dL	51 ± 13	54 ± 15	0.34
LDL Cholesterol, mg/dL	97 ± 26	91 ± 30	0.49
^a^Triglycerides, mg/dL	106 (69)	163 (106)	0.09
Glucose, mg/dL	90 ± 10	96 ± 14	0.08

Subjects self-reported whether they adhered to regular exercise (yes or no), with the level of exercise ranked as: 0, none; 1, 1 × week; 2, 2–4 × week; 3, > 5 × week

*BMI* body mass index, *LDL* low-density lipoprotein, *HDL* high-density lipoprotein

^a^Data reported as mean ± standard deviation or median with interquartile range

**Table 2:** HIV (+) subject immunologic and HAART related characteristics

Immunologic characteristic	Mean ± SD	Range
	Prevalence	
HIV History		
Length of HIV diagnosis, months	196 ± 99	48–360
Previous history of AIDS-defining diagnosis, %	25	---
Previous history of opportunistic infections, %	19	---
Undetectable viral load > 3 years, %	100	---
Compliance with HAART > 3 years, %	99	90–100
HAART-induced side effects		
Metabolic syndrome, %	22	---
Lipodystrophy, %	78	---
Lipoatrophy, %	63	---
Lipoaccumulation, %	67	---
HAART Exposure		
Cumulative HAART (any agent), months	157 ± 88	36–336
Cumulative exposure PI, months	91 ± 57	0–249
Cumulative exposure NRTI, months	163 ± 122	40–464
Cumulative exposure “high risk” PI, months	47 ± 33	0–103
Cumulative exposure NNRTI, months^a^	22 ± 63	0–156
Cumulative exposure INSTI, months^a^	5.5 ± 12.6	0–48
Cumulative exposure “high risk” NRTI, months^a^	44.2 ± 73.9	0–273
Immunologic History		
CD4+ cell count^a^	594 ± 326	242–2597
CD8+ cell count^a^	686 ± 356	312–1944
CD4+/CD8+ ratio^a^	78 ± 47	30–313

*PI* protease inhibitors, *NRTI* nucleoside reverse transcriptase inhibitors, *NNRTI* non-nucleoside reverse transcriptase inhibitors, *INSTI* integrase strand transfer inhibitors, *High risk PI’s* amprenavir, fosampenavir, indinavir, lopinavir, *High risk NRTI’s* abacavir, didanosine

^a^Data reported as mean + standard deviation or median with interquartile range

### Pericardial fat reproducibility

Pearson correlation coefficients for interobserver agreement were 0.98 (95 % CI 0.94–0.99, p < 0.0001) for pericardial fat volume at the level of LM origin, 0.9 (95 % CI 0.72–0.97, p < 0.0001) for pericardial fat volume at the level of RV free wall and 0.92 (95 % CI 0.77–0.97, p < 0.0001) for thickness of the pericardial fat at RV free wall.

### Pericardial fat and intramyocardial lipid content in HIV-positive and HIV-negative subjects

Pericardial fat volume at the level of the LM was significantly higher in HIV (+) subjects compared to HIV (-) controls (see Table 3). MR spectroscopy revealed a three-fold elevation in intramyocardial lipid content in HIV (+) subjects compared to controls (0.26 % vs. 0.85 %, *p* = 0.005).

**Table 3:** Pericardial fat measures and myocardial lipid content in HIV (+) subjects and HIV (-) controls

	HIV (+)	HIV (-)	P value
	*n* = 27	*n* = 22	
Pericardial fat			
Volume at LM origin, cm^3^	33.5 ± 12.5	27.5 ± 9.7	0.038
Volume at RV free wall, cm^3^	34.8 ± 12.4	33.4 ± 19	0.37
Thickness of pericardial fat at RV free wall, mm	10.9 ± 4.9	10.8 ± 8.1	0.48
Myocardial lipid content, %	0.85 ± 1	0.26 ± 0.26	0.006

### Pericardial fat predictors in all subjects

Pericardial fat volume at the level of LM origin showed strong correlation with FRS (*r* = 0.53, *p* = 0.0002), BMI (*r* = 0.71, *p* < 0.0001), waist circumference (*r* = 0.73, *p* < 0.0001), and waist/hip ratio (*r* = 0.7, p < 0.0001). No correlation was observed between pericardial fat volume at LM origin and family history (*r* = 0.06, *p* = 0.67), current (*r* = 0.16, *p* = 0.27)/previous (*r* = 0.23, *p* = 0.11) smoking or amount of exercise (*r* = 0.23, *p* = 0.1).

Pericardial fat volume at the level of RV free wall showed a weaker correlation with FRS (*r* = 0.44, *p* = 0.002), and no correlation was observed between FRS and thickness of pericardial fat at RV free wall (*r* = 0.29, *p* = 0.05). Correlation coefficients between pericardial fat measures and risk factors in all population, HIV (+) subjects and HIV (-) controls is detailed in Table 4.

**Table 4:** Pericardial fat volume and risk factors

	All	HIV (+)	HIV (-)
	r (p value)	r (p value)	r (p value)
BMI	0.71***	0.58***	0.79***
Waist circumference	0.73***	0.65**	0.77***
Hip circumference	0.39**	0.24 (0.23)	0.63**
Waist/hip ratio	0.70 ***	0.71**	0.60**
Family history	0.06 (0.67)	0.3 (0.13)	0.33 (0.14)
Current smoking	0.16 (0.27)	0.01 (0.94)	0.29 (0.18)
Prior smoking	0.23 (0.11)	0.1 (0.62)	0.25 (0.26)
Level of exercise	0.23 (0.1)	0.08 (0.68)	0.36 (0.09)
Total cholesterol	0.24 (0.1)	0.19 (0.36)	0.35 (0.12)
LDL cholesterol	0.23 (0.12)	0.10 (0.61)	0.33 (0.15)
Triglycerides	0.39**	0.40 (0.051)	0.64**
Fasting glucose	0.10 (0.49)	0.04 (0.82)	0.05 (0.8)
Risk factors for metabolic syndrome	0.48**	0.34 (0.09)	0.58**
Framingham risk score	0.53**	0.41*	0.77***

Correlation Coefficients between pericardial fat volume at the level of LM origin and risk factors in all population, HIV (+) subjects and HIV (-) controls

*BMI* body mass index, *LDL* low-density lipoprotein

*p < 0.05

**p < 0.01

***p < 0.0001

### Pericardial fat and intramyocardial lipid content in all subjects

**Fig. 1:**
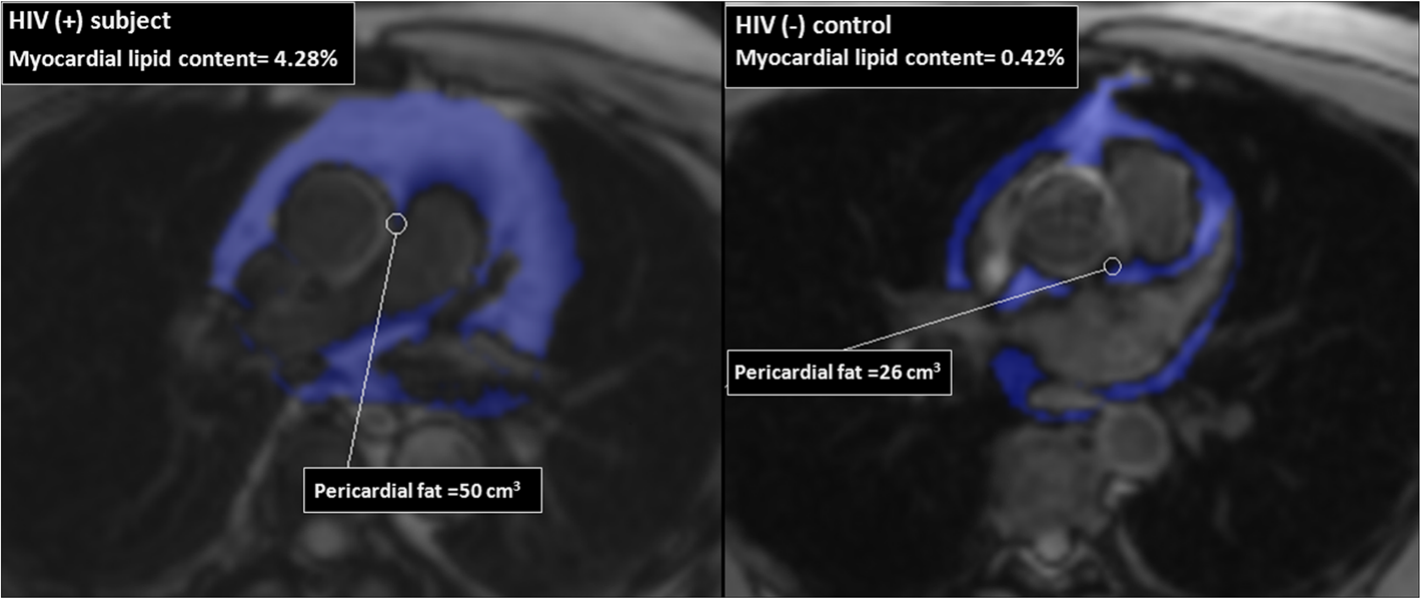
Pericardial fat volume quantification at level of the LM origin. On the *right*, 49 year old HIV (+) subject with BMI 24 kg/m^2^ and 27 years on HAART, quantification on CMR revealed a high pericardial fat volume and myocardial lipid content. On the *left*, 49 year old HIV (-) volunteer with BMI 29.5 kg/m2, quantification showed low pericardial fat volume and myocardial lipid content

Pericardial fat volume at the level of LM origin showed strong correlation with intramyocardial lipid content (*r* = 0.58, p < 0.0001, Fig. 1). Weaker correlation was found with pericardial fat volume at the RV free wall (*r* = 0.5, *p* = 0.0004) and thickness of pericardial fat at RV free wall (*r* = 0.44, *p* = 0.002).

### Multivariable analysis

Multivariable analysis adjusted for age, Framingham Risk Score (FRS) and waist/hip ratio, showed that only pericardial fat at the level of the LM origin was significantly associated to HIV-status (OR 1.09, 95 % confidence interval 1 - 1.2, *p* = 0.047), over FRS and waist/hip ratio. We also examined examined the relationship of pericardial fat, intramyocardial lipid content and lipoaccumulation with HIV-seropositivity. Intramyocardial lipid content was associated with HIV-seropositivity (*p* = 0.034, OR7.83 995 % CI: 1.2–52.4) but the association was not significant for either intramyocardial lipid content (*p* = 0.06) or pericardial fat (*p* = 0.45) when considered together. This was probably because the two measures are significantly correlated (Spearman’s Rank correlation coefficient 0.58, p < 0.0001). Prevalence of lipo-accumulation was significantly higher in HIV-seropositive subjects (18/23 or 78 %).

### Pericardial fat predictors in HIV-positive subjects

Pericardial fat volume at the level of LM origin showed significant correlation with time since HIV diagnosis (*r* = 0.5, *p* = 0.01) and duration of HAART exposure (*r* = 0.46, *p* = 0.02,). Pericardial fat volume at RV free wall showed a weaker correlation with time since HIV diagnosis (*r* = 0.45, *p* = 0.02) and no correlation with duration of HAART exposure (*r* = 0.38, *p* = 0.05). No correlation was observed between fat thickness at RV free wall and time since HIV diagnosis (*r* = 0.33, *p* = 0.1) or duration of HAART exposure (*r* = 0.26, *p* = 0.2).

Regarding specific HAART medication class exposure and pericardial fat volume at the level of LM origin, significant correlation was found only with current use of CCR5 receptor antagonists (*r* = 0.42, *p* = 0.03) and cumulative exposure of CCR5 receptor antagonists in months (*r* = 0.42, *p* = 0.03). Current NRTI use was negatively correlated (*r* = -0.42, *p* = 0.03).

Pericardial fat volume at the level of LM origin was significantly higher in subjects with lipo-accumulation (37 ± 13.3 cm3 vs. 27.1 ± 8.4 cm3, *p* = 0.03), but not in subjects with generalized lipodystrophy (35.6 ± 13.1 cm3 vs. 26.8 ± 8 cm3, *p* = 0.07) or lipoatrophy (33.5 ± 12.8 cm3 vs. 33.4 ± 12.7 cm3, *p* = 0.48). Pericardial fat volume at RV free wall was also significantly higher in patients with lipo-accumulation (38.2 ± 13.3 cm3 vs. 28.6 ± 7.6 cm3, *p* = 0.03), whereas pericardial fat thickness at RV free wall (11.5 ± 5.6 mm vs. 9.7 ± 3.6 mm, *p* = 0.19) was not significantly different.

## Discussion

HIV-related cardiovascular disease is an emerging contributor to morbidity and mortality. Imaging biomarkers such as pericardial or myocardial adiposity may serve as markers of cardiovascular risk in this population. Computed tomography is commonly used to quantify pericardial fat deposition; however, computed tomography remains limited by its need for ionizing radiation. CMR provides a safe, reliable means of serial cardiac evaluation without the hazards associated with ionizing radiation. In our study, we utilized CMR to demonstrate several important findings: first, that increased pericardial fat at the level of the LM origin, measured quickly and reproducibly from a standard CMR sequence, is significantly higher in HIV (+) patients treated with HAART. Second, this increase in pericardial fat is significantly associated with increased intramyocardial lipid content (i.e. cardiac steatosis). To our knowledge, this is the first report of this correlation of pericardial fat with cardiac steatosis. Third, we extend the existing literature [13, 14, 17] by showing that each of these markers are also associated with HIV history (i.e. years of infection), duration of HAART exposure, as well as the clinical presence of lipo-accumulation secondary to HAART. Remarkably, these results persist despite rigorous screening for cardiovascular risk factors in our HIV+ subjects, including family history of cardiovascular disease. That HAART exposure was found to be related to derangements in fat metabolism, reinforces the hypothesis that HAART itself contributes to cardiac alterations.

The exact mechanism responsible for pericardial fat deposition remains unclear. We found that HAART exposure was strongly associated with pericardial fat deposition, providing important mechanistic insight. Indeed, HAART is known to cause both lipodystrophy and hyperlipidemia [5–7] and in the present investigation, was associated with lipo-accumulation. Triglyceride infiltration into the myocardium has previously been associated with derangements in both diastolic relaxation and systolic contractility in both rodents [26–29] and humans [30, 31] One explanation for this observation is that ectopic fat accumulation contributes to the generation of lipotoxic intermediates, such as ceramide, which can trigger myocellular apoptosis [32]. Lipid vacuole infiltration can also dissemble the myocardial contractile apparatus, which could independently lead to contractile dysfunction [8, 33]. Thus, while our data cannot prove causality, we hypothesize that chronic exposure to HAART produces a metabolic-type derangement (clinically or non-clinically evident) leading to myocardial and pericardial fat accumulation. Taken together, we speculate that disturbances in the metabolic milieu, secondary to HAART exposure, leads to ectopic fat accumulation in HIV+ patients.

### Clinical relevance

Identification of multiple risk factors in the development of pericardial fat or myocardial triglyceride accumulation in HIV+ patients on HAART is paramount in developing clinical algorithms for appropriate cardiac screening and risk reduction implementation. Factors such as duration of HAART exposure, specific exposure to “cardiotoxic” antiretroviral agents, or overall duration of HIV infection may guide the clinician in screening maneuvers. Chronologic age alone cannot be the sole criteria for routine cardiovascular screening and assessment, as many individuals become infected with HIV at a relatively young age and have been on HAART for over a decade by the time they reach their early 30’s or 40’s. Thus, the concept of chronologic age as a cardiovascular screening indictor may be irrelevant, especially in younger HIV+ patients. As such, defining “HIV-infection-age” and “HAART-exposure” may be more informative to the clinician for CVD screening and assessment.

Ectopic fat deposition is emerging as an independent marker of cardiovascular risk in HIV-infected patients as demonstrated via computed tomography [34, 35]. In a study by Guaraldi et al. [14], epicardial adipose tissue was strongly associated with coronary artery calcium (an established marker of atherosclerosis) in patients with HIV infection on HAART. In our study, we show that pericardial fat deposition is related to HIV history, duration of HAART exposure, as well as the clinical presence of lipo-accumulation secondary to HAART. While these two fat depots are not entirely the same, both have been implicated in the development of coronary artery disease [36]. Further investigation is therefore warranted to determine whether pericardial fat content, measured by routine CMR, will be equally predictive for cardiovascular risk in HIV.

Although our study did not find specific protease inhibitors or nucleoside reverse transcriptase inhibitors (which are known to have cardiotoxic effects) to be more predictive of cardiac steatosis or pericardial fat accumulation, compared to other medications, the present investigation was not specifically powered to detect such differences. However caution must continue to be exercised when these medications are prescribed, and during routine CVD risk stratification.

Routine clinical qualitative screening for external lipodystrophy may serve as a useful screening tool for further CVD screening in this patient population. Indeed, this study utilized a straightforward method of evaluating lipodystrophy that can be performed by a general or subspecialist physician during a regular outpatient office consultation. Importantly, traditional indicators for CVD such as body mass index were not found to be associated with increased cardiac steatosis in HIV-infected patients on HAART, whereas waist/hip circumference ratio and lipodystrophy screening were found to be highly correlated with cardiac alterations.

### Limitations

There were several limitations to this study. First, our study population was small, however, both the patient and control groups were highly homogeneous with regard to multiple demographic parameters. Our selection process of HIV (-) control subjects closely approximated the HIV (+) subjects, therefore, the two study groups were very similar aside from HIV status and use of HAART. Second, our measurements provide information about pericardial fat and not exclusively epicardial fat, which has been proposed as the best marker for cardiovascular risk among thoracic fat deposits [8, 12]. Furthermore, our measurements of pericardial fat are obtained from a single slab in order to propose a practical fast approach to pericardial fat quantification, nevertheless a full volumetric assessment is more desirable and could be introduced in the future. Third, this was a cross-sectional study of HIV (+) patients on multiple HAART regimens, therefore, associations between specific classes of HAART and pericardial fat and intramyocardial lipid content were limited due to small groups of patients on particular medication regimens. Larger cohorts of HIV (+) patients on specific HAART regimens are necessary to determine medication class-induced risk profiles. This study did not include a group of HIV-infected patients without history of HAART exposure, thus preventing us from completely partitioning the independent role of HIV infection (without HAART) per se. In the United States, HAART is immediately implemented once the patient is able to make a decision to accept the risks and benefits of therapy, therefore, it would be extremely difficult to recruit a subset of HIV+ subjects without history of HAART. Finally, this study was limited to male subjects due to its sample size and significant reported differences in patient characteristics by gender among patients with HIV. As a result, these findings may not be generalizable to women, and future research in women is warranted. Studies in non-HIV-infected individuals have implicated pericardial fat accumulation with increasing cardiovascular risk and events [10–12]. Further studies are therefore warranted to investigate the relationship between pericardial fat accumulation and cardiovascular events in HIV-infected subjects.

## Conclusions

Our study show that pericardial fat, measured at the origin of LM, can be done both quickly and reproducibly and is highly correlated with intramyocardial lipid content, years of HIV infection, duration of HAART exposure and the clinical presence of lipo-accumulation. Pericardial fat by CMR therefore represents a viable biomarker for assessing cardiovascular risk in this population.

## References

[1] UN AIDS, global report. UN AIDS report on the global aids epidemic 2010. United Nations Press: United Nations AIDS Report; 2010.

[2] Hall HI, Song R, Rhodes P, Prejean J, An Q, Lee LM (2008). Estimation of HIV incidence in the United States. JAMA.

[3] Lewden C, May T, Rosenthal E, Burty C, Bonnet F, Costagliola D (2008). Changes in causes of death among adults infected by HIV between 2000 and 2005: the “Mortalite 2000 and 2005” surveys (ANRS EN19 and Mortavic). JAIDS.

[4] Varriale P, Saravi G, Hernandez E, Carbon F (2004). Acute myocardial infarction in patients infected with human immunodeficiency virus. Am Heart J.

[5] Mangili A, Jacobson DL, Gerrior J, Polak JF, Gorbach SL, Wanke C (2007). Metabolic syndrome and subclinical atherosclerosis in patients infected with HIV. Clin Infect Dis.

[6] Melzi S, Carenzi L, Cossu MV, Passerini S, Capetti A, Rizzardini G (2010). Lipid metabolism and cardiovascular risk in HIV-1 infection and HAART: present and future problems. Cholesterol.

[7] Caron M, Auclair M, Vissian A, Vigouroux C, Capeau J (2008). Contribution of mitochondrial dysfunction and oxidative stress to cellular premature senescence induced by antiretroviral thymidine analogues. Antivir Ther.

[8] Cheng VY, Dey D, Tamarappoo B, Nakazato R, Gransar H, Miranda-Peats R (2010). Pericardial fat burden on ECG-gated noncontrast CT in asymptomatic patients who subsequently experience adverse cardiovascular events. JACC Cardiovasc Imaging.

[9] Mahabadi AA, Massaro JM, Rosito GA, Levy D, Murabito JM, Wolf PA (2009). Association of pericardial fat, intrathoracic fat, and visceral abdominal fat with cardiovascular disease burden: the Framingham Heart Study. Eur Heart J.

[10] Ding J, Hsu FC, Harris TB, Liu Y, Kritchevsky SB, Szklo M (2009). The association of pericardial fat with incident coronary heart disease: the Multi-Ethnic Study of Atherosclerosis (MESA). Am J Clin Nutr.

[11] Dey D, Wong ND, Tamarappoo B, Nakazato R, Gransar H, Cheng VY (2010). Computer-aided non-contrast CT-based quantification of pericardial and thoracic fat and their associations with coronary calcium and metabolic syndrome. Atherosclerosis.

[12] Tamarappoo B, Dey D, Shmilovich H, Nakazato R, Gransar H, Cheng VY (2010). Increased pericardial fat volume measured from noncontrast CT predicts myocardial ischemia by SPECT. JACC Cardiovasc Imaging.

[13] Lo J, Abbara S, Rocha-Filho JA, Shturman L, Wei J, Grinspoon SK (2010). Increased epicardial adipose tissue volume in HIV-infected men and relationships to body composition and metabolic parameters. AIDS.

[14] Guaraldi G, Scaglioni R, Zona S, Orlando G, Carli G, Ligabue G (2011). Epicardial adipose tissue is an independent marker cardiovascular risj in HIV-infected patients. AIDS.

[15] Nelson MD, LaBounty T, Szczepaniak LS, Szczepaniak E, Smith L, St. John L (2014). Cardiac steatosis and left ventricular dysfunction is associated with exposure to Human Immunodeficiency Virus highly active antiretroviral therapy: a 3-Tesla cardiac magnetic resonance imaging study. J Am Coll Cardiol.

[16] Nelson MD, Victor RG, Szczepaniak EW, Simha V, Garg A, Szczepaniak LS (2013). Cardiac steatosis and left ventricular hypertrophy in patients with generalized lipodystrophy as determined by magnetic resonance spectroscopy and imaging. Am J Cardiol.

[17] Holloway CJ, Ntusi N, Suttie J, Mahmod M, Wainwright E, Clutton G (2013). Comprehensive cardiac magnetic resonance imaging and spectroscopy reveal a high burden of myocardial disease in HIV patients. Circulation.

[18] Alberti KGMM, Zimmet P, Shaw J (2005). The mteabolic syndrome -a new worldwide definition. Lancet.

[19] Wilson PWF, D'Agostino RB, Levy D, Belanger AM, Silbershatz A, Kannel WB (1998). Prediction of coronary heart disease using risk factor categories. Circulation.

[20] Oyama N, Goto D, Ito YM, Ishimori N, Mimura R, Furumoto T (2011). Single-slice epicardial fat area measurement: do we need to measure the total epicardial fat volume?. Jap J Radiol.

[21] Tran T, Small G, Cocker M, Yam Y, Chow BJW (2014). A single slice measure of epicardial adipose tissue can serve as an indirect measure of total apicardial adipose tissue burden and is associated with obstructive coronary artery disease. Eur Heart J Cardiovasc.

[22] Iacobellis G, Assael F, Ribaudo MC, Zappaterreno A, Alessi G, Di Mario U (2003). Epicardial fat from echocardiography: a new method for visceral adipose tissue prediction. Obes Res.

[23] Iacobellis G, Ribaudo MC, Assael F, Vecci E, Tiberti C, Zappaterreno A (2003). Echocardiographic epicardial adipose tissue is related to anthropometric and clinical parameters of metabolic syndrome: a new indicator of cardiovascular risk. J Clin Endocrinol Metab.

[24] Ahn SG, Lim HS, Joe DY, Kang SJ, Choi BJ, Choi SY (2008). Relationship of epicardial adipose tissue by echocardiography to coronary artery disease. Heart.

[25] Szczepaniak LS, Babcock EE, Schick F, Dobbins RL, Garg A, Burns DK (1999). Measurement of intracellular triglyceride stores by 1H spectroscopy: validation in vivo. Am J Endocrinol Metab.

[26] Nagarajan V, Gopalan V, Kaneko M, Angeli V, Gluckman P, Richards AM (2013). Cardiac function and lipid distribution in rats fed a high-fat diet: in vivo magnetic resonance imaging and spectroscopy. Am J Physiol Heart Circulation.

[27] Hankiewicz JH, Banke NH, Farjah M, Lewandowski ED (2010). Early impairment of transmural principal strains in the left ventricular wall after short-term, high-fat feeding of mice predisposed to cardiac steatosis. Circ Cardiovasc Imaging.

[28] Christoffersen C, Bollano E, Lindegaard ML, Bartels ED, Goetze JP, Andersen CB (2003). Cardiac lipid accumulation associated with diastolic dysfunction in obese mice. Endocrinology.

[29] Bakermans AJ, Geraedts TR, van Weeghel M, Denis S, João Ferraz M, Aerts JM (2011). Fasting-induced myocardial lipid accumulation in long-chain acyl-CoA dehydrogenase knockout mice is accompanied by impaired left ventricular function. Circ Cardiovasc Imaging.

[30] McGavock JM, Lingvay I, Zib I, Tillery T, Salas N, Unger R (2007). Cardiac steatosis in diabetes mellitus: a 1H-magnetic resonance spectroscopy study. Circulation.

[31] Hammer S, van der Meer RW, Lamb HJ, de Boer HH, Bax JJ, de Roos A (2008). Short-term flexibility of myocardial triglycerides and diastolic function in patients with type 2 diabetes mellitus. Am J Physiol Endrinol Metab.

[32] Szczepaniak LS, Victor RG, Orci L, Unger RH (2007). Forgotten but not gone: the rediscovery of fatty heart, the most common unrecognized disease in America. Circ Res.

[33] Borisov AB, Ushakov AV, Zagorulko AK, Novikov NY, Selivanova KF, Edwards CA (2008). Intracardiac lipid accumulation, lipoatrophy of muscle cells and expansion of myocardial infarction in type 2 diabetic patients. Micron.

[34] Orlando G, Guaraldi G, Zona S, Carli F, Bagni P, Menozzi M (2012). Ectopic fat is linked to prior cardiovascular events in men with HIV. J Acquir Immune Defic Syndr.

[35] Zona S, Raggi P, Bagni P, Orlando G, Carli F, Ligabue G (2012). Parallel increase of subclinical atherosclerosis and epicardial adipose tissue in patients with HIV. Am Heart J.

[36] Aslanabadi N, Salehi R, Javadrashid A, Tarzamni M, Khodadad B, Enamzadeh E (2014). Epicardial and pericardial fat volume correlate with the severity of coronary artery stenosis. J Cardiovasc Thor Res.

